# Chemokines and chemokine receptors: A new strategy for breast cancer therapy

**DOI:** 10.1002/cam4.3014

**Published:** 2020-04-06

**Authors:** Hui Liu, Zhenjiang Yang, Wenping Lu, Zhen Chen, Lianyu Chen, Shuyan Han, Xiaoyu Wu, Tiange Cai, Yu Cai

**Affiliations:** ^1^ College of Pharmacy Jinan University Guangzhou China; ^2^ Shenzhen Traditional Chinese Medicine Hospital Shenzhen China; ^3^ Guangan’ Men Hospital China Academy of Chinese Medical Sciences Beijing China; ^4^ Department of Integrative Oncology Cancer Center Fudan University Shanghai China; ^5^ Department of Integrative Oncology Shanghai Medical College Fudan University Shanghai China; ^6^ Department of Integration of Chinese and Western Medicine Key Laboratory of Carcinogenesis and Translational Research (Ministry of Education) Peking University Cancer Hospital & Institute Beijing China; ^7^ Leslie Dan Faculty of Pharmacy University of Toronto Toronto Canada; ^8^ College of Life Sciences Liaoning University Shenyang China; ^9^ Cancer Research Institute of Jinan University Guangzhou China; ^10^ International Cooperative Laboratory of Traditional Chinese Medicine Modernization and Innovative Drug Development of Chinese Ministry of Education (MOE) School of Pharmacy Jinan University Guangzhou China

**Keywords:** breast cancer, chemokine receptors, chemokines, therapeutic target

## Abstract

Chemokines and chemokine receptors not only participate in the development of tissue differentiation, hematopoiesis, inflammation, and immune regulation but also play an important role in the process of tumor development. The role of chemokines and chemokine receptors in tumors has been emphasized in recent years. More and more studies have shown that chemokines and chemokine receptors are closely related to the occurrence, angiogenesis, metastasis, drug resistance, and immunity of breast cancer. Here, we review recent progression on the roles of chemokines and chemokine receptors in breast cancer, and discuss the possible mechanism in breast cancer that might facilitate the development of new therapies by targeting chemokines as well as chemokine receptors. Chemokines and chemokine receptors play an important role in the occurrence and development of breast cancer. In‐depth study of chemokines and chemokine receptors can provide intervention targets for breast cancer biotherapy. The regulation of chemokines and chemokine receptors may become a new strategy for breast cancer therapy.

## Graphical abstract

This manuscript, comprehensively describes that the effects of chemokines and chemokine receptors on the occurrence, angiogenesis, metastasis, drug resistance and immune process of breast cancer. These findings provide a rationale for breast cancer therapy that target chemokines and chemokine receptors.

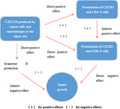

## 1 INTRODUCTION

Breast cancer is one of the most frequent cancers in the world, with a high incidence among women. Although surgical resection, chemotherapy, radiotherapy and molecular targeting can delay the progression of breast cancer, the prognosis is still poor. Metastasis and drug resistance are two important factors in increasing the mortality of breast cancer patients.^1^ The existing treatment methods have not achieved satisfactory clinical results, so it is urgent to find new strategies to effectively prevent the progression of breast cancer to improve the therapeutic effect. Chemokine is a kind of chemotactic cytokine, which drives lymphocytes, such as granulocytes, monocytes and macrophages, migrates in a specific way through specific binding with corresponding receptors, and participates in the process of human inflammatory response and immune regulation.^2^, ^3^ New evidence shows that chemokine is an important factor in the development of tumors. Chemokine receptors can initiate signal pathways that cause tumor proliferation, differentiation, invasion, and metastasis.^4^, ^5^ It has over expression in a variety of malignant tumors, such as breast cancer, lung cancer, ovarian cancer, and liver cancer tissues.^6^ Breast cancer cells can produce many chemokines, and express a variety of chemokine receptors, which play a wide role in the occurrence and progression of breast cancer. With the study of molecular mechanism of breast cancer, chemokines and chemokine receptors are expected to become a choice of new therapy for breast cancer. Here, we review the role of chemokines and chemokine receptors in breast cancer. These findings provide a rationale for developing therapies that target chemokines and chemokine receptors.

## 2 BREAST CANCER

According to the global cancer statistics,^7^ in 2018, there will be about 18.1 million new cancer cases and 9.6 million deaths worldwide. The incidence of breast cancer is the highest among female malignant tumors. It is estimated that more than 1 000 000 women are diagnosed with breast cancer every year, and more than 410 000 people will die of the disease.^8^ This trend suggests that breast cancer is an important world health issue. In addition to steroid hormones, other risk factors such as family genetics, age, gene mutation, and environmental pollution play an important role in the occurrence and development of breast cancer. At present, the main treatment mode of breast cancer is chemotherapy combined with surgery or radiotherapy. Chemotherapy is the main means of breast cancer systemic treatment, but the resistance of tumor cells to chemotherapy restricts the treatment effect. In addition, breast cancer often occurs in distant metastasis of bone, lung, brain, and liver, which seriously endangers women's physical and mental health and affects their quality of life. One of the characteristics of breast cancer is a highly inflammatory microenvironment supported by infiltrating immune cells, cytokines, and growth factors, which is conducive to the proliferation, migration, and invasion of cancer cells.^9^ Chemokines play an important role in the recruitment of stromal cells in tumor microenvironment.^10^ In the process of occurrence, progression and metastasis of breast cancer, the interaction between tumor cells and stromal cells should not be ignored, and the occurrence of this process cannot be separated from chemokines.

## 3 CHEMOKINES AND CHEMOKINE RECEPTORS

Chemokines are small molecular polypeptides produced by immune cells with molecular weight of 8‐10 KD, belonging to the cytokine superfamily. More than 50 chemokines have been found so far. These chemokines can be classified into CC, CXC, CX3C, and C subfamilies according to the number and location of N‐terminal cysteine molecules. In the chemokine family, CC chemokine represents the largest subgroup, including CCL1‐CCL28, with a wide chemotactic spectrum and chemotactic effects on monocytes, eosinophils/basophils, T lymphocytes, dendritic cells, etc. The CXC chemokine subfamily has 16 members including CXCL1‐CXCL16. According to the existence or absence of amino acid motifs composed of glutamic acid, leucine and arginine (EβLR motifs), CXC subfamily can be divided into CXC (ELR+) and CXC (ELR−). The CXC chemokine subfamilies are involved in tumor angiogenesis, and most ELR+ chemokines can directly induce endothelial cell activation to promote angiogenesis, while ELR− chemokines inhibit tumor angiogenesis. In addition, the members of C family chemokines are XCL1 and XCL2, while CX3C family chemokines currently have only one family member, CX3CL1. The former has obvious chemotaxis to T lymphocyte, B lymphocyte, and natural killer cell, while the latter belongs to membrane‐bound chemokines, which can chemotaxis monocyte and T lymphocyte. Chemokines, when combined with specific receptors of target cells, control various biological and pathological processes. A chemokine can bind to multiple chemokine receptors, and a chemokine receptor can also bind to multiple chemokines. Chemokine receptors belong to seven transmembrane G‐protein‐coupled receptor superfamily. They are not only expressed on macrophages, neutrophils, and other inflammatory cells but also on endothelial cells and some tumor‐derived epithelial cells.^11^ At present, there are no less than 20 chemokine receptors, which can be divided into CC receptor (CCR), CXC receptor (CXCR), CX3C receptor (CX3CR), and C receptor (CR) according to their ligands. In addition, although some chemokine receptors do not participate in cell migration or cell activation‐related cellular functions after binding with ligands, they can affect the availability and function of chemokines. These receptors are known as atypical receptors such as atypical chemokine receptor 1 (ACKR1), ACKR2, ACKR3, and ACKR4, which are also important molecules involved in health and disease processes.^12^, ^13^

When chemokines are combined with specific receptors, immune cells can be collected into tumor microenvironment to regulate immune surveillance, angiogenesis, invasion, and metastasis. To be sure, chemokines and chemokine receptors play a part in the carcinogenesis and development of cancer, but how to explain the regulatory mechanism clearly is still a problem to be solved. It has been found that chemokines can promote the proliferation and survival of cancer cells by different pathways. Different mechanisms of chemokines and chemokine receptors on tumor cells, such as induction of mitogen‐activated protein kinase/extracellular signal‐regulated kinase (ERK) signaling pathway, induction of expression of important growth stimulating genes cyclin D1,^14^ oncogene FOS,^15^ and human heparin‐binding epidermal growth factor (HB‐EGF),^16^ or increasing the expression of antiapoptotic gene MDM2.^17^ It also negatively regulates the expression of Bcl‐2 and the activation of Caspase‐3 and Caspase‐9^18^ to directly promote the proliferation of tumor cells. In addition, chemokines and chemokine receptors stimulate infiltrating leukocytes in tumor tissue to secrete IL‐10, transforming growth factor‐β (TGF‐β), matrix metalloproteinase, growth factor, and angiogenic factor to promote tumor growth, invasion, and immunosuppression, thus indirectly playing a role.^19^ Atypical chemokine receptor is a component of the regulatory network of cancer inflammation and immune system, which may play a role in tumorigenesis, depending on the type of cells it expresses and coexpression with other chemokine receptors.^20^, ^21^ In view of the critical role of chemokines and chemokine receptors in the occurrence, development and metastasis of cancer, more and more attention has been paid to chemokines and chemokine receptors in the study of recurrence, metastasis, and prognosis of cancer.

## 4 THE ROLE OF CHEMOKINES/CHEMOKINE RECEPTORS IN BREAST CANCER

### 4.1 Chemokines/chemokine receptors and tumor growth

As the first stage of malignant evolution, chronic inflammation is an important cause of breast cancer.^22^ Studies have shown that chronic inflammation can increase the risk of breast cancer, and breast cancer cells can secrete inflammatory regulatory factors to promote the progression of inflammation, thus forming a vicious cycle of inflammation‐tumors to accelerate cancer progression.^23^ The inflammatory mediators produced by the inflammatory response can lead to the formation of nonspecific proinflammatory cytokines (IL‐1α/β, IL‐6, IFN‐α, and tumor necrosis factor‐α [TNF‐α]), which in turn can induce chemokine expression to promote inflammatory.^24^ This study showed that chemokines and chemokine receptors in inflammatory microenvironment can help to maintain tumor growth. Clinical studies have shown that some chemokines are upregulated in breast cancer, which is closely related to tumor growth. For example, the levels of CCL5 increase significantly in plasma of most types of breast cancer.^25^ Lapteva and his colleagues have found that the proliferation rate of basal‐like breast cancer cells that inhibit CXCR4 expression by small interfering RNA technology is significantly lower than that of normal breast cancer cells, which provides direct evidence for the key role of CXCR4 in the growth of breast cancer cells.^12^ ACKR3, an atypical chemokine, is also overexpressed in many types of cancer, and is involved in tumor proliferation and angiogenesis. It was found that the expression of ACKR3 at protein and mRNA levels in precancerous breast cancer was much higher than that in adjacent and normal breast tissues.^26^ Several studies have shown that the high expression of ACKR3 is positively correlated with the proliferation of breast cancer cells, indicating that ACKR3 has a positive effect on tumor growth.^27^, ^28^, ^29^ Furthermore, Boudot and his colleagues found that in estrogen receptor (ER) positive breast cancer, estradiol can promote the growth of ER positive breast cancer cells by producing a large number of CXCL12 and regulating CXCR4 and CXCR7 receptors of CXCL12, and the chemokine receptor CXCR4 can enhance the response of cells to estradiol.^30^ Therefore, other factors are also involved in the growth of breast cancer through chemokines and chemokine receptors, which indirectly shows that they are important participants in the tumor growth process.

Tumor growth is the result of multiple factors, chemokines and chemokine receptors are the key molecules in this process. The biological axis of CXCR4 and its receptor CXCL12 (Stromal cell‐derived factor‐1) promotes cancer cell growth, invasion and metastasis in most tumors including breast cancer.^31^, ^32^ In addition, it has been found that fibroblast‐derived CXCR4 promotes breast cancer growth and is associated with poor prognosis.^33^ Most studies have proved that chemokines or chemokine receptors can promote the growth of breast cancer, but how they play a role in the growth process of tumor cells is one of the goals of basic tumor researchers. New research found that in most types of breast cancer patients, high levels of CCL5 in plasma promote the expression of CX3CL1 to promote breast cancer cell proliferation through epidermal growth factor signal transduction pathway, and also promote epithelial‐mesenchymal transition.^34^ Recent research has shown that the overexpression of CCL28, a member of CC family chemokines, can not only significantly enhance the proliferation of breast cancer cells but also effectively inhibit apoptosis.^35^ Min Yao and her colleagues have found that the growth of primary tumors increased significantly with the cotransplantation of fibroblasts and the expression of CCL2 was high in a murine model of basal‐like breast cancer.^36^ In addition, bone marrow‐derived CCL5 could promote the growth of triple negative breast cancer by regulating the production of myeloid‐derived suppressor cells (MDSCs),^37^ which indicated that it may indirectly promote the growth of breast cancer by influencing the recruitment of tumor‐related immune cells. Hypoxia is a major factor in promoting tumor growth, which is closely related to cancer progression.^38^, ^39^, ^40^ It was found that the overexpression of CCR5 can increase cell migration, while knockout of CCR5 can reduce hypoxia‐mediated cell migration. And further research found that the high expression of hypoxia‐inducible factor‐1 (HIF‐1) mRNA was related to the levels of CCR5 mRNA and CCL5 mRNA in clinical samples.^13^ The results showed that HIF‐1 was involved in the regulation of CCR5 and CCL5 under hypoxia. CCL5 and tumor‐derived colony‐stimulating factor work together to promote the production of MDSCs in bone marrow, which helps to maintain the growth of breast cancer.^41^ These results suggest that chemokines and chemokine receptors interact with other factors in tumor microenvironment to maintain the growth of breast cancer cells.

### 4.2 Chemokines/chemokine receptors and tumor angiogenesis

Tumor neovascularization plays a key role in the development of malignant tumors, including the malignant transformation of cells, clonal proliferation of transformed cells, local invasion, and distant metastasis.^42^ Angiogenesis can provide oxygen and nutrients for tumor growth, and can timely remove and transport the metabolic products produced by the tumor, so that the tumor can continue to develop. In addition, angiogenesis helps the invasion of malignant cells to promote tumor metastasis. There are abundant blood vessels around the cancerous tissue in breast cancer patients, which provides favorable conditions for tumor growth and metastasis.^43^ More studies further proved that the occurrence, invasion, and metastasis of breast cancer depend on the formation of tumor neovascularization.^44^, ^45^ Tumor angiogenesis factors are regulated by both angiogenic factors and angiogenesis inhibitors, and chemokines and their receptors play an important role. CXC family ELR+ chemokines such as CXCL (1, 2, 3), CXCL5, CXCL6, CXCL8 can effectively promote tumor angiogenesis. Most ELR− chemokines stabilize blood vessels and inhibit angiogenesis by inhibiting chemotaxis to endothelial cells, such as CXCL4, CXCL9, and CXCL10.^46^ Chemokines mutated by ELR motifs or adding ELR motifs to CXC‐ELR− chemokines can promote angiogenesis, suggesting that ELR motifs are essential for the angiogenesis/angiogenesis inhibitory activity of CXC chemokine.^47^ It is worth mentioning that although CXCL12 belongs to ELR− chemokine, but it can promote tumor angiogenesis.^48^ Also, under the stimulation of hypoxia and angiogenic factor,^49^ the secretion and aggregation of CXCL12 in the tumor tissues increased. On the one hand, it can significantly promote the secretion of vascular endothelial growth factor, which can enhance the proliferation and migration of endothelial cells. On the other hand, CXCL12 can enhance the activation of endothelial cells by increasing the expression of intercellular adhesion molecule‐1.^50^ The expression of several angiogenesis molecules including vascular endothelial growth factor and chemokine CXCL8 is also regulated by the transcription of hypoxia‐inducible factors under hypoxia.^51^, ^52^, ^53^

Lin and her colleague have found that CCL18 released by tumor‐associated macrophages (TAMs) could promote angiogenesis and tumor progression in breast cancer. At the same time, they confirmed that CCL18 and vascular endothelial growth factor synergistically promoted endothelial cell migration and angiogenesis in vitro and in vivo.^54^ CCL5 can directly promote the production of MMP‐9 in breast cancer cells, which indicates that the relationship between chemokines and matrix metalloproteinases may be of great significance to angiogenesis.^55^ In addition, chemokines can enhance angiogenesis by recruiting immune cells. For example, TAMs promote tumor angiogenesis by producing various angiogenic factors such as VEGF, TGF‐β, MMP‐2, and MMP‐9.^56^ However, chemokines CCL2 and CCL8 can further promote tumor growth and angiogenesis by recruiting and activating tumor‐related macrophages.^57^, ^58^ CXCL6 also promotes angiogenesis through recruitment of centralized granulocytes.^59^ In addition, atypical chemokine receptors are also involved in tumor angiogenesis. Cancer cells and endothelial cells can regulate cell invasion, adhesion, and angiogenesis by activating AKT dependent expression of atypical receptor ACKR3/CXCR7.^60^, ^61^ These results suggest that chemokines can directly promote tumor angiogenesis, but also play a role in tumor angiogenesis through a variety of ways. Certainly, chemokines can not only promote the development of breast cancer, but also inhibit it. Thomas and his colleagues have found that CXCL14 can effectively inhibit tumor angiogenesis, thereby reducing tumor cell migration.^62^ In addition, the study showed that CXCL14 transgenic mice had a significant inhibitory effect on cancer cell transplanted tumors.^63^ Gu and her colleagues found that the overexpression of CXCL14 inhibited the proliferation and invasion of breast cancer cells, and weakened the growth and lung metastasis of xenograft tumors. It should be noted that the mechanism may be related to the inhibition of angiogenesis.^64^ Therefore, CXCL14 can inhibit the growth and metastasis of breast cancer cells, suggesting that CXCL14 is an anticancer chemokine in breast cancer.

### 4.3 Chemokines/chemokine receptors and tumor metastasis

Tumor angiogenesis provides the basis for tumor metastasis, and tumor metastasis is an important feature of malignant tumors and the main cause of death in patients with advanced breast cancer.^65^ The metastasis of breast cancer involves three steps: first, some tumor cells undergo local infiltration, intravascular transport, and extravasation to form micrometastasis. Then other cancer cells acquire this cloning ability and carry out micrometastasis. Finally, micrometastasis develops into visible metastasis and leads to breast cancer recurrence and distant organ metastasis. The process of metastasis from primary tumors to metastases is rigorous rather than random.^66^ Metastasis is a dynamic process in which cancer cells spread from primary tumors to distant organs, but this process is nonrandom and selective. More and more studies have shown that chemokines and chemokine receptors are involved in the process of metastasis. For example, the expression of CCL19 and CCL21, ligands of CCR7, was significantly increased in lymph nodes of breast cancer patients. In addition, the expression level of CCL21 is higher than CCL19, which indicates that the interaction between CCL21 and CCR7 in breast cancer may be better than that CCL19.^67^, ^68^ It has been found that CCR7 is highly expressed in triple negative breast cancer cell lines and breast cancer tissues, and when CCR7 is absent, it can significantly reduce the proliferation, migration, and invasion of triple negative breast cancer cells.^69^ In addition, the activation of CCL5 can promote breast cancer metastasis; insulin‐like growth factor 1‐mediated mammary gland cancer cell migration is shown to be dependent on the CCL5‐CCR5 interaction.^70^

**FIGURE 1:**
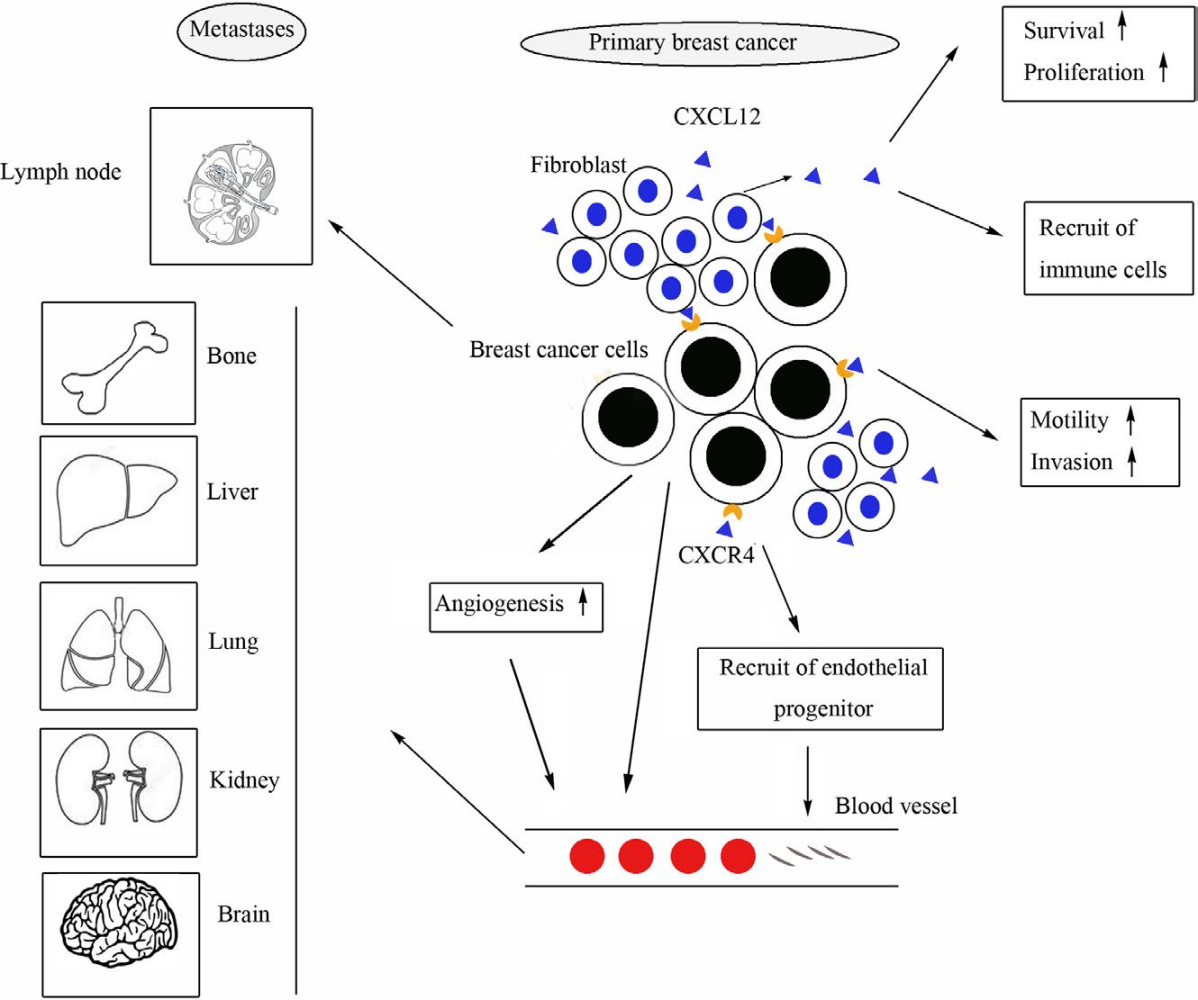
The role of CXCL12/CXCR4 in breast cancer. CXCR4 expressed in breast cancer cells can promote the growth of primary tumors by binding specifically to CXCL12 secreted by tumor‐associated fibroblasts. CXCL12 secreted by tumor microenvironment not only promotes the proliferation and survival of cancer cells but also recruits immune cells and bone marrow‐derived cells to migrate to tumor micrsoenvironment. The expression of CXCR4 promotes the metastasis of tumor cells to specific sites such as bone, liver, lung, brain, lymph node, and kidney

Obviously, these highly expressed tumor chemokines and chemokine receptors have a significant role in promoting the metastasis of breast cancer cells, and may play a particularly important role in tumor progression (Figure 1). It was further found that in a murine model of triple negative breast cancer, CCR7 knockout reduced the metastasis of 4T1 cells. The results demonstrated that the expression of CCR7 in triple negative breast cancer was related to tumor metastasis. The possible mechanism was that epithelial cells, adhesion molecules, and other signal pathways participated in tumor metastasis.^71^ Zhang and his colleague have found that CCL5 deficiency significantly reduces both primary tumor load and lung metastasis in PYMT gene mouse mammary gland virus model (MMTV‐PYMT) transgenic mice with lumen breast cancer and this effect is related to Th2 cell deficiency.^72^ This suggests that chemokines and chemokine receptors promote tumor metastasis by establishing related mechanisms. Chemokines can activate many related pathways to conduct signal transduction in cells to promote the progression of tumor malignant transformation. The chemokine CXCL10 and its receptor CXCR3 play a role in breast cancer metastasis and osteoclast activation. It was found that the secretion of CXCL10 gradually increased during the growth of 4T1 cells in vitro, and CXCL10/CXCR3 axis formed a positive feedback loop through a typical NF‐kB signaling pathway.^73^ These results suggest that the NF‐kB signaling pathway mediated by CXCL10/CXCR3 may play a role in controlling the autonomic regulation of CXCL10 and the malignant characteristics of 4T1 cells. Interleukin‐8 (IL‐8) is an important molecule in regulating breast cancer metastasis. Some studies have shown that CXCR2, as an IL‐8 receptor, may promote breast cancer migration, invasion, and metastasis by promoting the migration of breast mesenchymal stem cells^74^ or mediate bone metastasis of breast cancer.^75^ Clinical data analysis showed that the high expression of CXCR2 was related to the high expression of cyclooxygenase 2 (COX2) and the low expression of P85A, AKT1 or E‐cadherin, and B‐catenin in cancer tissues.^76^ Further studies have indicated that CXCR2 promoted breast cancer metastasis by inhibiting AKT1 and activating COX2.^77^

In addition, in the process of metastasis of breast cancer cells, including macrophages, fibroblasts, and other tumor‐related cells are recruited to the metastasis focus, creating favorable environmental conditions for the metastasis process. Chemokine and chemokine receptor promote tumor cell metastasis by influencing the infiltration and recruitment of immune cells and tumor‐related cells. For example, ACKR2 prevents natural killer cells from infiltrating into tumor tissue by limiting the expression of CCR2, and ultimately supports metastasis.^78^ CCL2 mediated the recruitment of macrophages expressing CCR2+ to promote metastasis has become an established mechanism^79^; CCR2 overexpression can increase the survival rate and invasion rate of AUW225 breast cancer, which may be related to the accumulation of fibroblasts expressing CCL2.^80^ In the study of the relationship between tamoxifen resistance and cancer‐associated fibroblasts, it was found that after TAM treatment, the secretion of CXCL16 increased under the guidance of G‐protein coupled receptor 30, and the exocrine CXCL16 promoted the migration and invasion of MCF‐7 cells.^81^

### 4.4 Chemokines/chemokine receptors and tumor drug resistance

The diversity and complexity of tumor often induce the resistance of tumor cells to chemotherapy drugs, which reduces the sensitivity of tumor cells to therapeutic drugs, and further mediates the recurrence and metastasis of breast cancer, eventually leading to death. In recent years, targeted therapy has greatly improved the treatment of breast cancer. For example, chemotherapy combined with trastuzumab has significantly reduced the mortality rate of Her2‐positive breast cancer, and has become the first‐line treatment strategy for Her2‐positive breast cancer patients.^82^ However, due to the complexity of breast cancer and the uniqueness of targeted therapy, the resistance of tumor cells to targeted therapy remains an obstacle to cancer treatment. SRC, an intracellular tyrosine kinase, which plays a key role in controlling cell signaling pathways related to cell proliferation and survival. It has been found that its activity is significantly increased in breast, colon, liver, and other cancers. The combination of SRC inhibitors and anti‐HER2 therapy is effective against primary tumors, but acquired resistance of SRC inhibitors to tumor cells remains a therapeutic impediment. Fang and her colleagues have found that highly expressed plasminogen activator inhibitor‐1, an anti‐SRC inhibitor, could promote resistance to SRC inhibitors by inducing the secretion of CCL5.^83^ Crystal and his colleagues have showed that the interaction between CCR9 and CCL25 could resist the death of cancer cells induced by cisplatin. It was further found that CCR9 could promote the proliferation of tumor cells proliferation and upregulate the anti‐apoptosis mediated by the PI3K/AKT pathway.^84^ Another study found that CXCL12/CXCR4 signal axis activated HER2 through SRC, promoting the growth and proliferation of breast cancer cells, so that MCF‐7 cells have resistance to tamoxifen.^85^ Therefore, chemokines and chemokine receptors play an important role in the process of drug resistance. The mechanism of chemokines and chemokine receptors regulating drug resistance of breast cancer is still in further study, but it is worth to be sure that chemokine and chemokine receptor are related to drug resistance of breast cancer.

### 4.5 Chemokines/chemokine receptors and tumor immunity

**FIGURE 2:**
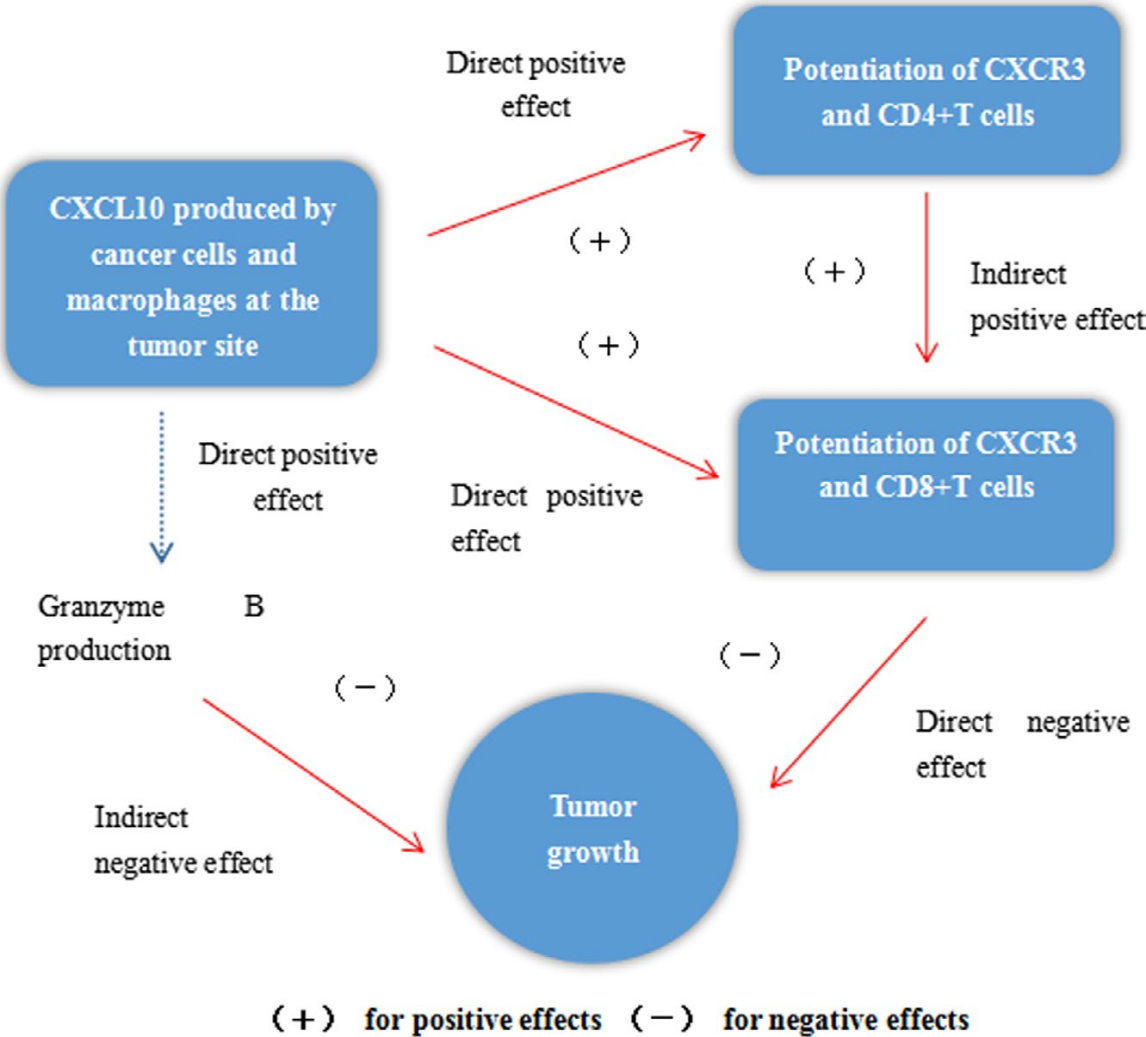
The antitumor activity of CXCL10. CXCL10 can inhibit the growth of tumor cells with direct antitumor activity, but also play an indirect antitumor role. On the one hand, CXCL10, which is produced in large quantities in tumors, indirectly enhances its antitumor activity by recruiting CXCR3, CD4+ T, and CD8+ T cells to tumor sites. CXCL10, on the other hand, inhibits tumor growth by inducing Granzyme B production

Breast cancer cells and stromal cells, including tumor‐infiltrating lymphocytes (TILs), constitute the microenvironment of breast cancer and play an important role in the whole development process of breast cancer. In tumor microenvironment, TIL plays a two‐way role in tumor development: on the one hand, they attack and kill tumor cells to inhibit tumor progression; on the other hand, they screen tumor cells that are more suitable for survival in immunoactive host or change tumor microenvironment, and ultimately promote tumor progression.^86^ CD4+ T cells, Th1 cells and effector CD8+ T cells are the key drivers of tumor immunity in TIL. Tumor‐related immune cells, endothelial cells, and fibroblasts constitute a complex tumor microenvironment, which is involved in the occurrence and development of breast cancer. The interaction between chemokines and chemokine receptors can recruit different subsets of immune cells into the tumor microenvironment. TIL is a key component in the process of tumor immunity, and the degree of TIL infiltration is closely related to the prognosis of patients. CD4+ T cells, Th1 cells, and effector CD8+ T cells are key drivers of antitumor immunity. Recent studies have shown that CXCL10 can enhance the antitumor activity of Th1 cells by inducing the recruitment of CXCR3 and CD8+ T cells to tumor sites and the production of Granzyme‐B induced by these cells.^87^ The results showed that CXCL10 could enhance the antitumor immunity by acting on CD4 and CD8+ T cells. The antitumor activity of CXCL10 at the tumor site is shown in Figure 2. In addition, a new subgroup (CCR8, CD4+ T cells, Foxp3+ Treg cells) was found, which is a key factor in promoting immune regulation. CCL1, one of CCR8 ligands, can induce CCR8, Foxp3, CD39, Granzyme‐B, and IL‐10 to enhance their inhibitory activity. Therefore, antitumor immunity can be promoted by blocking the interaction between CCR8 and CCL1.^88^ The effect of ACKR1 on chemokines is related to the expression environment, and its expression on erythrocytes maintains the homeostasis of chemokines, thereby isolating chemokines in the circulation, while expression on endothelial tissue transfers chemokines from the tissue to the circulation. Brittany D and his colleague have studied the differential gene expression of ACKR1 in mammary epithelial tumor tissues by IHC method and found that it is related to the unique characteristics of immune cell infiltration and related proinflammatory chemical factors.^89^ Studies have shown that the expression of ACKR1 is increased, and the level of circulating and infiltrating CCL2 is higher in tumors, while the level of CXCL8 (IL‐8) is lower. It is also related to T cells, B cells, macrophages, and monocytes, thus showing the characteristics of tumor‐infiltrating immune cells (CTLs) in breast cancer patients expressing ACKR1.^90^ It is suggested that the expression of ACKR1 in breast epithelial tumor can affect the infiltration of immune cells in TME, and ultimately affect the response of tumor to immunotherapy. Besides, early‐stage breast cancer has dense fibrous stroma and immunosuppressive effect. Metastatic breast cancer, such as liver and lung tends to be highly fibrotic and lacks cytotoxic T lymphocytes. CXCL12/CXCR4 axis is highly expressed in human primary and metastatic tumors. Inhibiting CXCR4 signal transduction can not only reduce fibrosis but also increase CTL infiltration and reduce immunosuppression in a murine model of metastatic breast cancer. The results showed that CXCL12/CXCR4 axis promoted immunosuppression by increasing fibrosis of tumor cells. At present, immunosuppressive strategies can reduce the incidence and mortality of tumors, and pebuprozumab is effective in the treatment of triple negative breast cancer. New studies show that chemokines enhance the efficacy of pebuprozumab in the treatment of triple negative breast cancer^91^ by regulating the expression of CTLs and PD‐1/PD‐L1/PD‐L2 in tumors. The results also indicate the importance of chemokines and chemokine receptors in tumor immunity. To be sure, the interaction of chemokines and specific receptors recruits different immune cell subsets to participate in the tumor immune process in the tumor microenvironment, so as to play a role in promoting or inhibiting tumor.

These results indicate that chemokines and chemokine receptors are closely related to tumor growth, angiogenesis, metastasis, drug resistance, and immunity in breast cancer. Therefore, the thorough study of chemokines and chemokine receptors provide new directions for breast cancer therapy. We summarize the effects of chemokines and chemokine receptors on breast cancer in Table 1.

**TABLE 1:** Chemokines/chemokine receptors function in breast cancer

	Receptor(s)	Type of studies	Possible mechanisms	Function on breast cancer	References
CCL2	CCR2	In vitro and in vivo	Recruit CAFs	Promote growth and invasion	^80^
		In vivo	Recruit monocytes, NKT cells and monocytic MDSCs	Promote proliferation, stemness and survival	105, 106
CCL5	CCR5	In vivo	Induce Th2 polarization in CD4+ T Cells	Promote metastasis	^72^
		In vitro and in vivo	Regulate MDSCs	Promote growth	^13^
		In vitro and in vivo	Promote the production of MMP9	Promote angiogenesis	^56^
CCL2/CCL8	Not mentioned	In vitro and in vivo	Recruit and activate TAMs	Promote growth and angiogenesis	58, 59
CCL18	ACKR1	In vitro and in vivo	Infiltration of TAMs, activate ERK and AKT/GSK‐3β/Snail signaling in HUVECs	Promote angiogenesis	^55^
		In vitro	Via PITPNM3 regulation	Promote invasion and metastasis	^107^
CCL19/CCL21	CCR7	In vitro and in vivo	Epithelial cells, adhesion molecules, and other signaling pathways	Promote proliferation and metastasis	68, 69, 70
CCL25	CCR9	In vitro	Enhance the expression of MMP‐1, ‐9, ‐11, and ‐13 active proteins	Promote migration and invasion	^108^
		In vitro and in vivo	PI3K/AKT Pathway	Promote growth and inhibit apoptosis	^84^
CCL28	Not mentioned	In vitro and in vivo	Via Bcl‐2 regulation	Promote growth and inhibit apoptosis	^36^
CXCL1	CXCR2	In vitro and in vivo	ERK/MMP‐2/9 signaling axis	Stimulate migration and invasion	^109^
CXCL6	Not mentioned	In vitro and in vivo	Recruit NK cells	Promote angiogenesis	^60^
CXCL8	CXCR2	In vitro and in vivo	Promote migration of MSC	Promote migration or bone metastasis	74, 75
CXCL10	CXCR3	In vitro	NF‐kB signaling pathway	Promote bone metastasis	^73^
CXCL12	CXCR4 CXCR7	In vitro and in vivo	Via E2	Promote growth, invasion, and migration	^31^
		In vitro and in vivo	Activation of HER2 by Src protein	Promote growth, proliferation, and drug resistance	^85^
CXCL16	Not mentioned	In vitro	Mediated by GPR30	Promote migration and invasion	^81^
CX3CL1	CX3CR1	In vitro and in vivo	EGF pathway	Promote proliferation and EMT	^35^
Not mentioned	ACKR1	In vitro	Tumor infiltrating immune cells	Indirect effects on proliferation and invasion	^90^
Not mentioned	ACKR2	In vitro and in vivo	Prevent NK cells infiltration	Promoting metastasis	^78^
Not mentioned	ACKR3 CXCR7	In vitro	EKT pathway	Regulate of cell invasion, adhesion, and angiogenesis	60, 61

Abbreviations: ACKR1, atypical chemokine receptor 1; EGF, epithelial‐mesenchymal transition; ERK, extracellular signal‐regulated kinase; HUVEC, human umbilical vein endothelial cells; MDSCs, myeloid‐derived suppressor cells; MSC, mesenchymal stem cell; NKT, natural killer T; PITPNM3, PITPNM family member 3; TAM, tumor‐associated macrophages.

## 5 CHEMOKINES/CHEMOKINE RECEPTORS AND CANCER THERAPY

Breast cancer is considered as a heterogeneous disease, characterized by different biological and phenotypic features which make its diagnosis and treatment challenging. The chemokine network composed of chemokine and chemokine receptor plays a complex role in the occurrence and development of breast cancer. Some chemokines can inhibit the growth of breast cancer cells, and some chemokines become "helpers" of breast cancer cells, which promote the malignant evolution of tumors. For example, CCL2, CCL5, CXCL8, and CXCL12 can promote breast cancer,^92^, ^93^, ^94^, ^95^, ^96^ while CXCL9, CXCL10, and CCL16 can inhibit breast cancer.^97^, ^98^, ^99^, ^100^ More and more researchers realize that chemokine research provides a new way for the treatment of breast cancer and other malignant tumors, and the related research with chemokine as the target for cancer is also in progress. The positive expression rate of CXCR4 in breast cancer is as high as 60%. It has been confirmed that CXCR4 is involved in lung metastasis of breast cancer.^91^, ^97^, ^101^ Therefore, drugs targeting CXCR4 may bring good results for breast cancer treatment. More chemokines have been found in breast cancer, which provides more choices for target therapy of chemokines. The inhibition of CXCL13 expression resulted in significant decrease of IL‐1, TNF‐α, and TGF‐β1, decrease of cell proliferation, and increase of apoptotic rate. The antitumor effect is due to the inhibition of ERK signal in breast cancer cells.^102^ Data from this study suggest that CXCL13 inhibition may be a potential way to block the progression of breast cancer. High expression of CCR5 and its cognate ligands (CCL4, CCL5) in breast cancer promote tumor progression. Asim and his colleagues have found that blocking CCR5 expression not only promotes the proliferation and apoptosis of metastatic breast cancer cells but also significantly inhibit bone metastasis in nude mice implanted with MDA‐MB‐231 cells.^103^ The results suggest that CCR5 may be a potential therapeutic target for advanced breast cancer. Tumor recurrence is also one of the obstacles of tumor treatment. More than half of breast cancer‐related deaths are caused by tumor recurrence after treatment. Residual tumor cells survive after treatment. The interaction between tumor microenvironment and tumor recurrence is very important. The unique environment of tumor microenvironment makes the residual tumor cells produce immunosuppression, and a large number of tumor‐related immune cells are recruited to participate in the process of tumor recurrence, so as to promote the continuous development and even malignant evolution of tumor. Andrea and her colleagues have found that CCL5 promotes recurrence of breast cancer by recruiting macrophages expressing CCR5, which may contribute to the accumulation of residual tumors, so blocking the tumor necrosis factor α‐CCL5 macrophage axis may be one of the effective ways to prevent recurrence of breast cancer.^104^ We summarized the drug research targeting chemokine/chemokine receptor in breast cancer in Table 2.

**TABLE 2:** Drug studies targeting chemokines/chemokine receptors in breast cancer

Target	Inhibitor	Results	References
CCR2	CCX9588 + anti‐PD‐L1	Reduce the number of MDSCs and inhibit growth and inhibit lung metastasis	^110^
CCR5	Maraviroc	Inhibit bone metastasis	^103^
CCL2	CNTO 888 + radiotherapy	Promote angiogenesis and metastasis	^111^
CXCR2	CXCR2^−/−^ + PTX (Table 3)	Reduce growth, angiogenesis, and inhibit lung metastasis	^105^
CXCR4	Reparixin + PTX	Reduce the number of MDSCs and inhibit metastasis	^112^
	Balixafortide + Eribulin	Inhibit metastasis	^113^
	GST‐NT2 1MP	Decrease growth, adhesion, migration, and reduce metastasis	^114^
	AMD3465	Inhibit growth and metastasis	^115^
CXCL12‐CXCR7	LYG202	Inhibit activation of endothelial cells and angiogenesis	^116^

Abbreviation: MDSCs, myeloid‐derived suppressor cells.

**TABLE 3:** Abbreviation

Abbreviation	Full fight
MAPK	Mitogen‐activated protein kinase
ERK	Extracellular signal‐regulated kinase
HB‐EGF	Human heparin‐binding epidermal growth factor
TGF‐β	Transforming growth factor‐β
ER	Estrogen receptor
TNF‐α	Tumor necrosis factor‐α
SDF‐1	Stromal cell‐derived factor‐1
EGF	Epidermal growth factor
EMT	Epithelial‐mesenchymal transition
MDSCs	Myeloid‐derived suppressor cells
HIF‐1	Hypoxia‐inducible factor‐1
ICAM‐1	Intercellular adhesion molecule‐1
TAMs	Tumor‐associated macrophages
IGF‐1	Insulin‐like growth factor 1
MMTV‐PYMT	PYMT gene mouse mammary gland virus model
IL‐8	Interleukin‐8
NF‐kB	Nuclear factor kappa beta
COX2	Cyclooxygenase 2
CAFs	Cancer‐associated fibroblasts
GPR30	G‐protein coupled receptor 30
NK	Natural killer cell
PAI‐1	Plasminogen activator inhibitor‐1
TIL	Tumor‐infiltrating lymphocytes
Tregs	Regulatory cells
CTLs	Tumor‐infiltrating immune cells
NKT	Natural killer T
Th2	Helper T cell 2
HUVECs	Human umbilical vein endothelial cells
PITPNM3	PITPNM family member 3
MSC	Mesenchymal stem cell
HER2	Human epidermal growth factor receptor‐2
E2	Estradiol
PTX	Paclitaxel

In addition, chemokines and chemokine receptors can also be used as effective biomarkers for early diagnosis and prognosis of breast cancer. The results showed that the levels of CCL2 and CCR2 were significantly higher in breast cancer patients, which could be used as a molecular marker for breast cancer diagnosis. Clinical data showed that the expression of CXCR4 was related to the overall survival rate in invasive patients. Reza and his colleagues found that upregulation of the CXCL12/CXCR4 chemokine axis in invasive breast cancer samples compared with normal adjacent lesions.^105^ It is suggested that chemokine and chemokine receptor may be biomarkers to predict the early diagnosis and prognosis of breast cancer.

## 6 SUMMARY AND PROSPECT

Chemokines and chemokine receptors play an important role in many physiological and pathological processes, and also have an important impact on the occurrence and development of breast cancer. We review the role of chemokines and chemokine receptors on the occurrence, angiogenesis, metastasis, drug resistance, and immune process of breast cancer, and explore the possible mechanism of promoting the development of new therapies for breast cancer by targeting chemokines or chemokine receptors. Chemokine receptors are used as drug targets to inhibit the growth and metastasis of tumor cells by blocking chemokine signal transduction by developing chemokine receptor inhibitors, chemokine modifiers, and neutralizing antibodies. In the future, chemokines and chemokine receptors may not only be the intervention target of tumor biotherapy but also be the biomarker to assist the early diagnosis and prognosis of breast cancer. Drug regulation of chemokine and chemokine receptors provides a new direction for the treatment of breast cancer. However, due to the large number of chemokine family members, the interaction between them is complex. Therefore, using chemokine or chemokine receptor as a single target may have some limitations, and the strategy of multi‐target regulation of chemokine network is more conducive to the treatment of breast cancer. In addition, the combination of targeted chemokine drugs and chemotherapeutic drugs may have a better therapeutic effect on breast cancer. With the further study of the interaction mechanism of chemokine and chemokine receptors with breast cancer, we can further understand the pathogenesis and development process of breast cancer, and provide new strategies for breast cancer therapy.

## CONFLICT OF INTEREST

The authors declare no competing interests.

## AUTHOR CONTRIBUTION

Hui Liu, Zhenjiang Yang, and Wenping Lu designed and wrote the manuscript. Zhen Chen and Lianyu Chen contributed to the revision and drafted Figures 1 and 2. Shuyan Han, Xiaoyu Wu, and Tiange Cai revised the sections in their expertise. Yu Cai supervised and finalized the manuscript. All authors reviewed the manuscript and approved the final version.
